# The Association Between Brain Temperature and Neurological Outcome in Out-of-Hospital Cardiac Arrest Patients Who Received Targeted Temperature Management at 33 °C

**DOI:** 10.31083/RCM43855

**Published:** 2025-11-18

**Authors:** Seok Jin Ryu, Byung Kook Lee, Dong Hun Lee, Yong Hun Jung, Kyung Woon Jeung, Wan Young Heo

**Affiliations:** ^1^Department of Emergency Medicine, Chonnam National University Medical School, 61469 Gwangju, Republic of Korea; ^2^Department of Emergency Medicine, Chonnam National University Hospital, 61469 Gwangju, Republic of Korea

**Keywords:** cardiac arrest, neurological outcomes, brain temperature, targeted temperature management

## Abstract

**Background:**

Despite the established concordance between core temperature and brain temperature (BT) in out-of-hospital cardiac arrest (OHCA) patients, the relationship between BT and neurological outcomes in those who received targeted temperature management (TTM) has yet to be elucidated. Thus, this study aimed to explore the relationship between BT and neurological outcome in OHCA patients who received TTM.

**Methods:**

This observational study involved adult patients (≥18 years) with OHCA who received TTM at 33 °C between April 2021 and December 2023. We recorded BTs at the initiation of TTM (BT_INI_) and during the maintenance phase of TTM (BT_MAIN_). A neurological outcome at 6 months was the primary outcome. Poor outcome was considered as Cerebral Performance Categories 3, 4, and 5.

**Results:**

Of the 149 included patients with OHCA, 109 (73.2%) patients exhibited poor outcomes. Compared with the good outcome group, the BT_INI_ (35.8 °C [interquartile range (IQR), 33.4–36.3 °C] vs. 33.4°C [IQR, 32.6–35.4 °C]) and BT_MAIN_ (33.1 °C [IQR, 32.8–33.2 °C] vs. 32.6 °C [IQR, 32.2–32.9 °C]) were lower in the poor outcome group. Multivariate analysis after adjusting for confounders revealed that BT_INI_ (odds ratio (OR), 0.223; 95% confidence interval (CI), 0.054–0.917; *p* = 0.038) and BT_MAIN_ (OR, 0.078; 95% CI, 0.019–0.322; *p* < 0.001) were associated with poor outcomes.

**Conclusions:**

BTs at the initiation of TTM and during the maintenance phase of TTM at 33 °C are associated with poor outcomes.

## 1. Introduction

Out-of-hospital cardiac arrest (OHCA) is a major contributor to global morbidity and mortality, and many survivors experience significant neurological deficits despite intensive resuscitation care following the return of spontaneous circulation (ROSC) [1, 2, 3]. Thus, accurate prediction of neurological outcomes in OHCA patients helps guide treatment and supports effective resource allocation [4, 5].

Among studies related to neurological outcomes after ROSC, studies conducted on core body temperature (CT) have shown that hypothermia on admission is associated with poor neurological outcomes [6, 7]. Post-rewarming fever after targeted temperature management (TTM) may also contribute to poor prognosis in OHCA patients after ROSC [8, 9]. However, CT primarily reflects systemic physiological states and may not accurately represent cerebral thermal dynamics or the extent of hypoxic-ischemic brain injury [10]. In contrast, brain temperature (BT) is directly influenced by cerebral metabolic activity and regional blood flow, making it a more direct indicator of brain status [11, 12]. Yablonskiy *et al*. [13] demonstrated a strong correlation between BT changes and oxidative metabolism using functional magnetic resonance imaging. Similarly, Wang *et al*. [10] reported that BT is fundamentally dependent on the balance between metabolic heat production and heat dissipation. In patients with subarachnoid hemorrhage, a BT higher than CT has been associated with preserved mitochondrial function and improved neurological outcomes [14]. However, previous studies on temperature in OHCA have mostly focused on CT as a predictor of neurological outcomes [6, 7, 8, 9]. There have been no clinical studies specifically investigating the relationship between BT and neurological outcomes in patients with OHCA.

Therefore, the purpose of this study was to evaluate the association between BT and neurological outcomes in adult OHCA survivors. We hypothesized that lower BT during TTM would be correlated with poorer neurological outcomes, potentially reflecting the severity of hypoxic-ischemic brain injury.

## 2. Materials and Methods

### 2.1 Study Design and Population

This prospective observational study utilized data from adult comatose OHCA survivors who were treated with TTM at Chonnam National University Hospital in Gwangju, Korea, between April 2021 and December 2023. The study was approved by the Institutional Review Board of Chonnam National University Hospital. Written informed consent was secured from all patients or their legal guardians before inclusion.

Adult (≥18 years) cardiac arrest patients receiving TTM were included in the study. Patients whose TTM was discontinued due to death or transfer, patients whose target body temperature was not 33 °C, or patients with missing CT or BT records were excluded.

### 2.2 TTM and Temperature Management During TTM

Survivors of comatose cardiac arrest who received TTM according to the guidelines maintained a target body temperature of 33 °C for 24 hours using an Arctic Sun® feedback-controlled surface cooling device (Energy Transfer Pads™; Medivance Corp, Louisville, CO, USA). Following completion of the TTM maintenance phase, rewarming was performed at 0.25 °C/hour until 36.5 °C. CT was assessed using an esophageal temperature probe. BT was measured using a zero-heat-flux sensor system (3M™ Bair Hugger™370, Saint Paul, MN, USA) attached to the center of the forehead [15, 16]. We collected CT and BT every hour from the initiation to the end of TTM.

### 2.3 Data Collection and Primary Outcome

We obtained the following data from hospital records: sex, age, preexisting illness, bystander cardiopulmonary resuscitation (CPR), witnessed collapse, etiology of cardiac arrest, presence of initial shockable rhythm, interval from collapse to ROSC, serum glucose, lactate, partial pressure of oxygen, and partial pressure of carbon dioxide (PaCO_2_) levels after ROSC. We recorded CTs and BTs at the initiation of TTM (CT_INI_ and BT_INI_) and during the maintenance phase of TTM (CT_MAIN_ and BT_MAIN_).

We examined neurological outcomes at 6 months after ROSC via a phone interview using the Cerebral Performance Category (CPC) scale. The scoring was as follows: 1 = good performance, 2 = moderate disability, 3 = severe disability, 4 = vegetative state, and 5 = brain death or death [17]. The primary outcome was a poor neurological outcome, defined as CPC 3–5. Telephone interviews were conducted using a structured algorithm comprising six hierarchical questions designed to systematically determine CPC scores while simultaneously assessing the Modified Rankin Scale (mRS) (**Supplementary Fig. 1**). Trained research personnel documented all responses on standardized case report forms, which were retained as part of the study records to ensure reproducibility.

### 2.4 Statistical Analysis

Categorical variables are reported as frequencies and proportions, while continuous variables are presented as medians with interquartile ranges because they did not pass the test for normality. Categorical variables between groups were analyzed using chi-squared tests with continuity correction for 2 × 2 contingency tables. For categorical variables, those with small expected cell counts less than 5 were analyzed using Fisher’s exact test. The Mann–Whitney U test was used to compare continuous variables between groups.

To assess the association between temperature variables and poor outcomes, multivariable logistic regression analysis was performed. We performed collinearity diagnostics with multivariable analysis. Variables exhibiting *p* < 0.20 in univariable comparisons were included in the multivariable regression model. A backward stepwise selection approach was employed to construct the final adjusted regression model, sequentially removing variables with *p* > 0.10, in accordance with a previously published methodology [18]. The elimination process was terminated when all remaining variables had *p*-values < 0.10 (**Supplementary Table 1**). Results from the logistic regression analysis are expressed as odds ratios (ORs), accompanied by 95% confidence intervals (CIs). An area under the receiver operating characteristic curve (AUROC) analysis was performed to examine the prognostic performance of temperature variables (continuous variables) for poor outcomes. We calculated the area under the curve (AUC), sensitivity, specificity, positive predictive value (PPV), and negative predictive value (NPV), which are presented along with 95% CIs. Cut-off values maximizing diagnostic performance were selected according to Youden’s index [19]. Post-hoc power analysis was conducted using G*power software (version 3.1.7, Heine Heinrich University, Düsseldorf, Germany).

All analyses were performed using PASW/SPSS™ software, version 26.0 (IBM Corp., Armonk, NY, USA) and MedCalc version 23.0 (MedCalc Software, bvba, Ostend, Belgium). Statistical significance was defined at a two-sided significance level of 0.05.

## 3. Results

### 3.1 Patient Characteristics

**Fig. 1.**
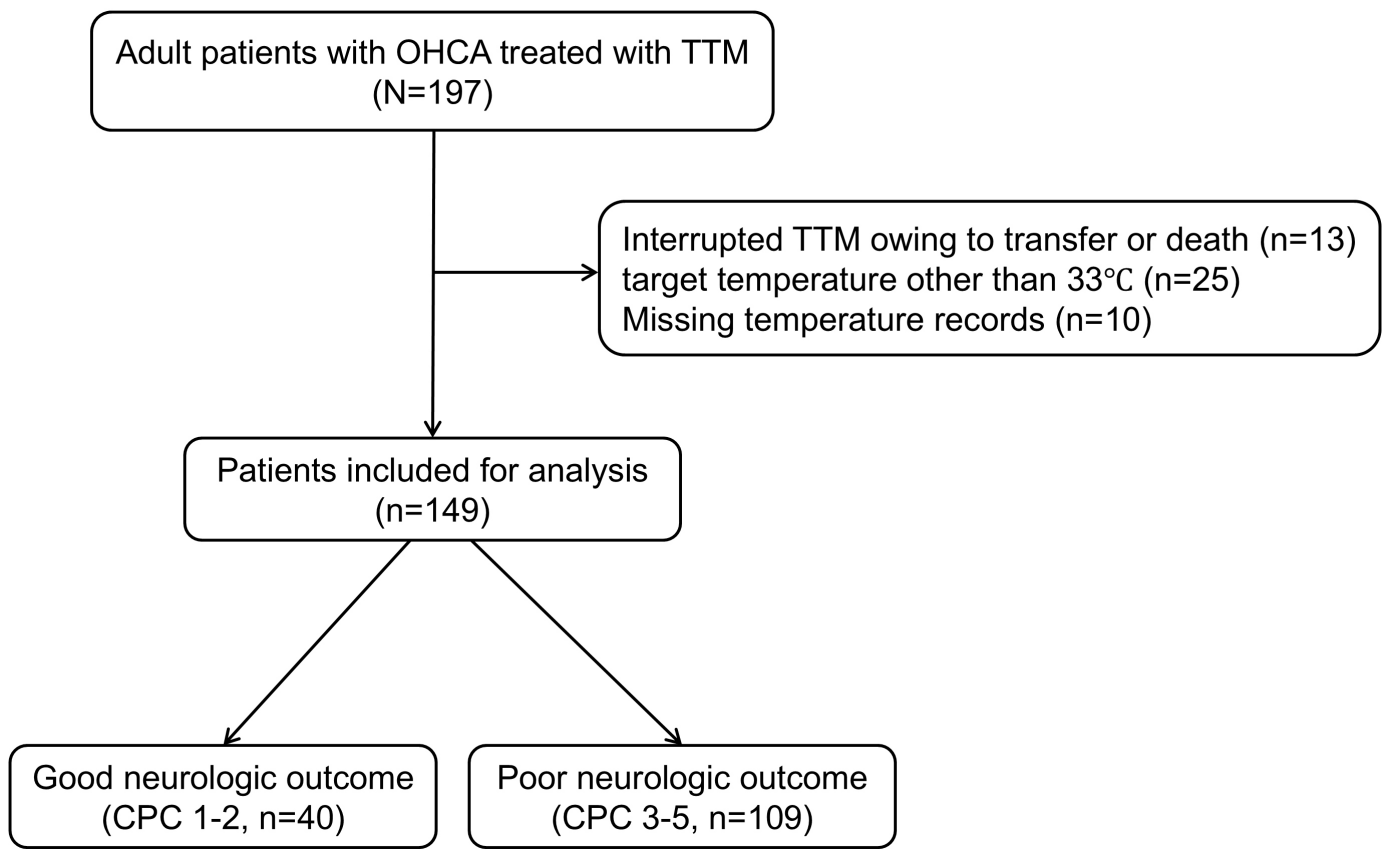
**Schematic diagram illustrating the number of patients included in the study**. OHCA, out-of-hospital cardiac arrest; TTM, targeted temperature management; CPC, Cerebral Performance Category.

We treated a total of 197 OHCA patients who received TTM over the study period. Of the total, 149 patients satisfied the inclusion criteria, as depicted in Fig. 1. The median age of the patients was 62 (48.5–71.0) years. There were 109 (73.2%) patients with poor outcomes.

Table 1 presents the baseline characteristics stratified by neurological outcomes. In comparison with patients with good outcomes, those with poor outcomes were older (62.0 vs. 57.5 years, *p* = 0.017) and had a higher incidence of diabetes (42.2% vs. 15.0%, *p* = 0.004), a lower incidence of shockable rhythm (25.7% vs. 85.0%, *p* < 0.001) and cardiac etiology (45.9% vs. 85.0%, *p* < 0.001), and a longer interval from collapse to ROSC (36.0 vs. 19.5 min, *p* < 0.001). Following ROSC, they had higher serum lactate (9.3 vs. 5.3 mmol/L, *p* < 0.001), glucose (268 vs. 219 mg/dL, *p* = 0.011), and PaCO2 (51.0 vs. 39.0 mmHg, *p* < 0.001) levels.

**Table 1.** **Baseline characteristics stratified by poor neurological outcomes at 6 months**.

Variables		Total (N = 149)	Good (N = 40)	Poor (N = 109)	*p*
Demographics					
	Age, years	62.0 (48.5–71.0)	57.5 (38.3–65.0)	62.0 (50.5–72.0)	0.017
	Male, n (%)	110 (73.8)	30 (75.0)	80 (73.4)	1.000
Preexisting illness, n (%)					
	Coronary artery disease	20 (13.4)	5 (12.5)	15 (13.8)	1.000
	Hypertension	79 (53.0)	19 (47.5)	60 (55.0)	0.527
	Diabetes	52 (34.9)	6 (15.0)	46 (42.2)	0.004
	Renal impairment	21 (14.1)	3 (7.5)	18 (16.5)	0.193
Cardiac arrest characteristics					
	Witnessed collapse, n (%)	92 (61.7)	29 (72.5)	63 (57.8)	0.148
	Bystander CPR, n (%)	85 (57.0)	27 (67.5)	58 (53.2)	0.169
	Shockable rhythm, n (%)	62 (41.6)	34 (85.0)	28 (25.7)	<0.001
	Cardiac etiology, n (%)	84 (56.4)	34 (85.0)	50 (45.9)	<0.001
	Time to ROSC, min	32.0 (20.0–45.0)	19.5 (14.3–26.8)	36.0 (26.5–47.5)	<0.001
Clinical characteristics after ROSC					
	Lactate, mmol/L	8.0 (5.0–11.9)	5.3 (3.0–8.0)	9.3 (5.9–13.3)	<0.001
	Glucose, mg/dL	250 (179–319)	219 (151–285)	268 (188–335)	0.011
	PaO_2_, mmHg	180.0 (103.1–268.5)	204.5 (115.1–341.0)	171.0 (100.0–247.0)	0.094
	PaCO_2_, mmHg	45.0 (36.0–60.0)	39.0 (31.5–41.8)	51.0 (38.0–66.8)	<0.001

CPR, cardiopulmonary resuscitation; ROSC, return of spontaneous circulation; PaO_2_, partial pressure of oxygen; PaCO_2_, partial pressure of carbon dioxide.

### 3.2 Comparison of Temperature Variables Stratified by Neurological Outcomes

**Fig. 2.**
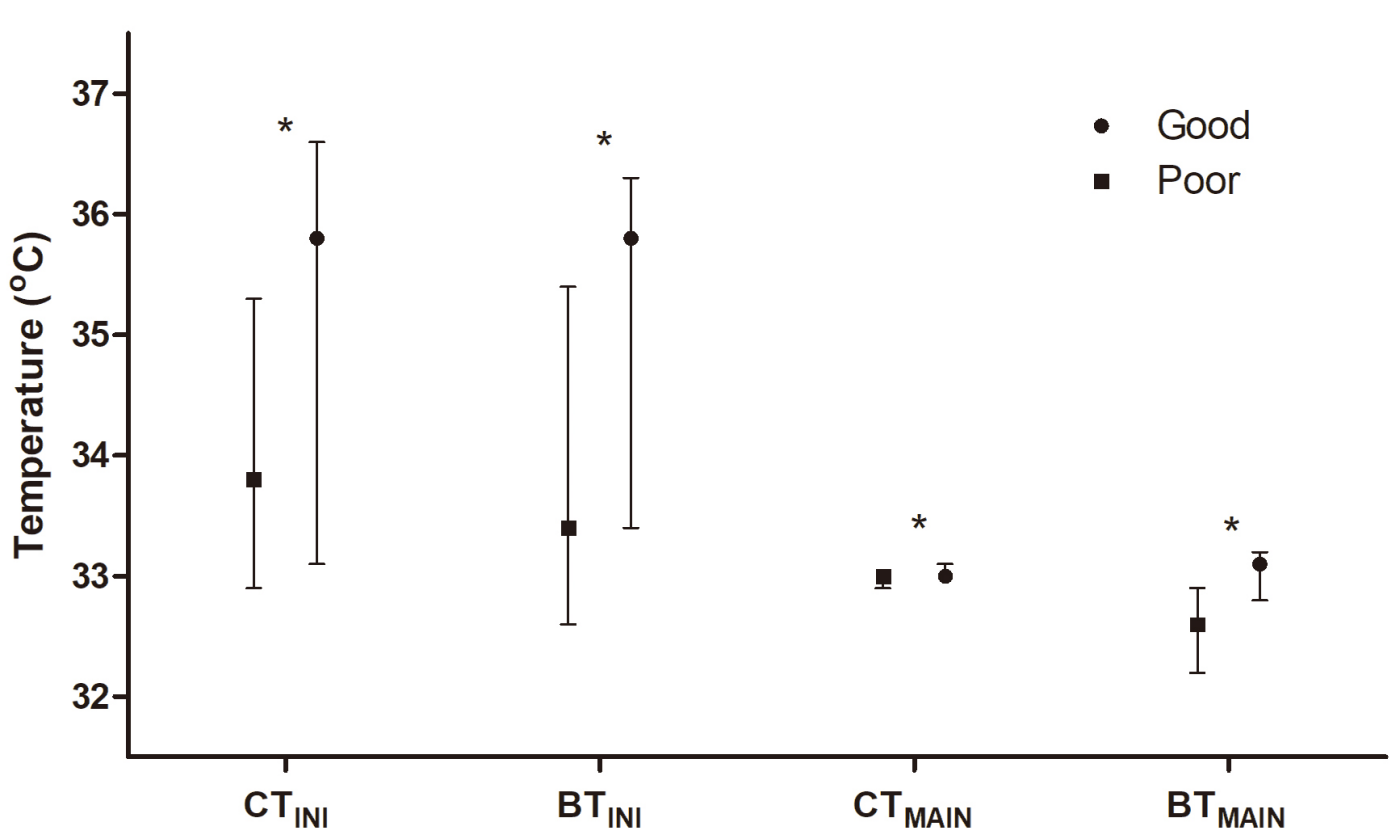
**Comparison of core and brain temperatures at the initiation (CT_𝐈𝐍𝐈_ and BT_𝐈𝐍𝐈_) and at the maintenance phase (CT_𝐌𝐀𝐈𝐍_ and BT_𝐌𝐀𝐈𝐍_) of targeted temperature management according to neurological outcomes**. The asterisk (*) indicates *p* < 0.05.

Fig. 2 shows temperature variables stratified by neurological outcomes. Significant differences were observed in CT_INI_ (interquartile range (IQR), 35.8 °C [33.1–36.6 °C] vs. 33.8°C [IQR, 32.9–35.3 °C], *p* < 0.001) and CT_MAIN_ (33.0 °C [IQR, 33.0–33.1 °C] vs. 33.0 °C [IQR, 32.9–33.0 °C], *p* = 0.030) between patients with good and poor outcomes. BT_INI_ (35.8 °C [IQR, 33.4–36.3 °C] vs. 33.4 °C [IQR, 32.6–35.4 °C], *p* < 0.001) and BT_MAIN_ (33.1 °C [IQR, 32.8–33.2 °C] vs. 32.6 °C [IQR, 32.2–32.9 °C], *p* < 0.001) were lower in patients with poor outcomes than in patients with good outcomes. Post-hoc power analyses of BT_INI_ and BT_MAIN_ yielded high statistical power values (0.99) for both variables.

### 3.3 Association Between Temperature Variables and Poor Neurological Outcomes

After adjusting for potential confounders, BT_INI_ (OR, 0.223; 95% CI, 0.054–0.917; *p* = 0.038) and BT_MAIN_ (OR, 0.078; 95% CI, 0.019–0.322; *p* < 0.001) were independently associated with poor outcomes (Table 2). However, CT_INI_ and CT_MAIN_ were not associated with poor outcomes in multivariable analysis.

**Table 2.** **Multivariable logistic regression analysis of brain and core temperatures during TTM for poor neurological outcomes at 6 months**.

Variables	Adjusted OR (95% CI)	*p*
CT_INI_, °C	3.193 (0.860–11.857) ^a^	0.083
BT_INI_, °C	0.223 (0.054–0.917) ^a^	0.038
CT_MAIN_, °C	0.239 (0.000–282.824) ^b^	0.692
BT_MAIN_, °C	0.078 (0.019–0.322) ^a^	<0.001

TTM, targeted temperature management; OR, odds ratio; CI, confidence interval; CT_INI_, core temperature at initiation of TTM; BT_INI_, brain temperature at initiation of TTM; CT_MAIN_, core temperature at maintenance phase of TTM; BT_MAIN_, brain temperature at maintenance phase of TTM. ^a^Adjusted for age, shockable rhythm, interval from collapse to return of spontaneous circulation, lactate level, and glucose level. ^b^Adjusted for age, diabetes, shockable rhythm, interval from collapse to return of spontaneous circulation, lactate level, glucose level, PaCO_2_ level.

**Fig. 3.**
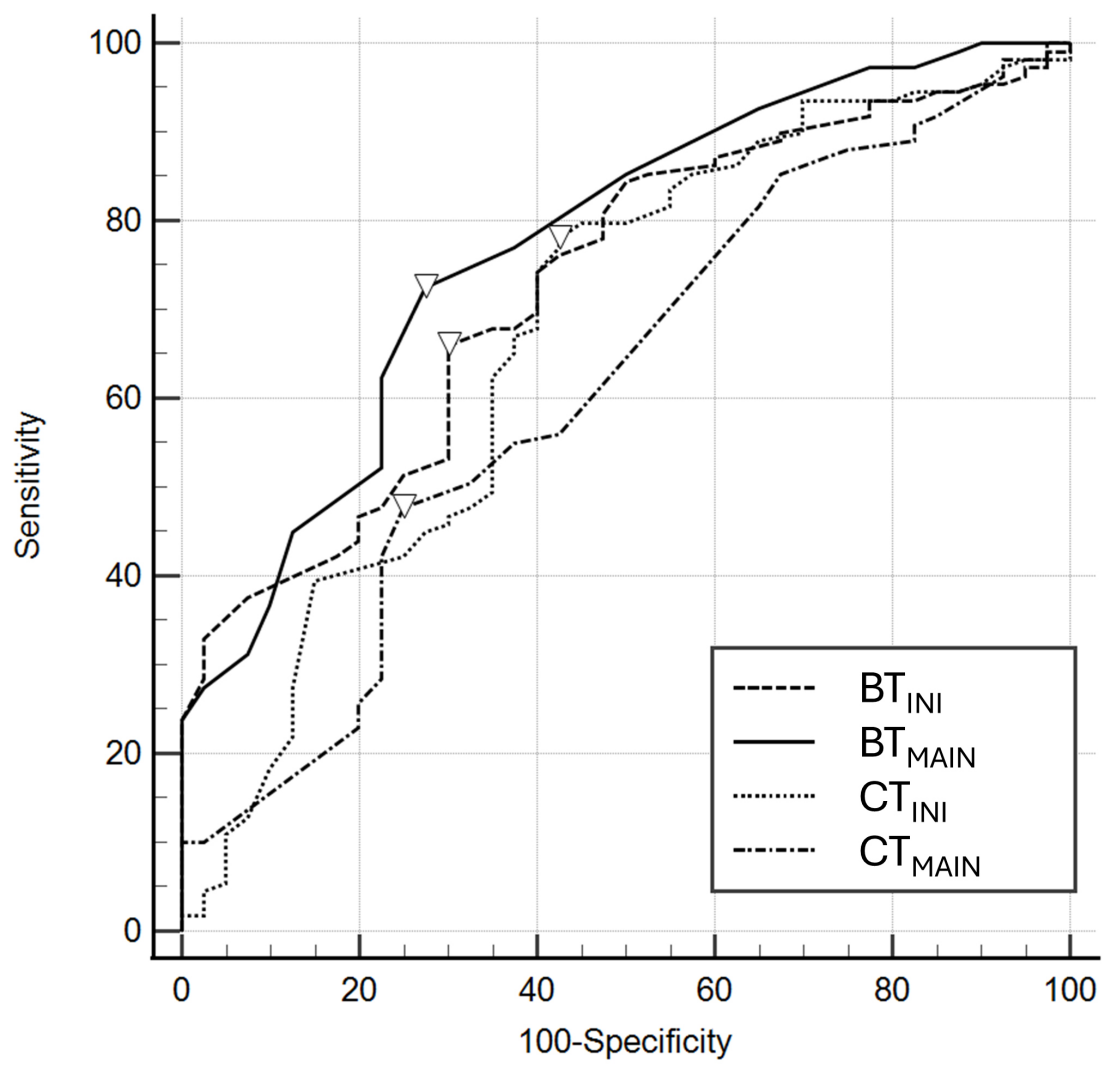
**Receiver operating characteristics of core and brain temperatures at the initiation (CT_𝐈𝐍𝐈_ and BT_𝐈𝐍𝐈_) and at the maintenance phase (CT_𝐌𝐀𝐈𝐍_ and BT_𝐌𝐀𝐈𝐍_) for poor neurological outcomes**. The inverted triangles on the curve are cutoff values ​​by Youden’s index.

Table 3 and Fig. 3 show the results of AUROC analysis of temperature variables for predicting poor outcomes. The AUCs of CT_INI_ and CT_MAIN_ were 0.677 (95% CI, 0.596–0.751) and 0.615 (95% CI, 0.532–0.693), respectively. The AUCs of BT_INI_ and BT_MAIN_ were 0.728 (95% CI, 0.649–0.797) and 0.773 (95% CI, 0.697–0.838), respectively.

**Table 3.** **AUC analysis of brain and core temperatures during TTM for poor neurological outcomes at 6 months**.

Variable	AUC (95% CI)	*p*	Cut-off value	Sensitivity (95% CI)	Specificity (95% CI)	PPV (95% CI)	NPV (95% CI)
CT_INI_, °C	0.677 (0.596–0.751)	<0.001	≤35.4	78.0 (69.0–85.4)	57.5 (40.9–73.0)	83.3 (77.5–87.9)	48.9 (38.1–59.0)
BT_INI_, °C	0.728 (0.649–0.797)	<0.001	≤34.3	66.1 (56.4–74.9)	70.0 (53.5–83.4)	85.7 (78.6–90.8)	43.1 (35.2–51.3)
CT_MAIN_, °C	0.615 (0.532–0.693)	0.028	≤32.96	47.7 (38.1–57.5)	75.0 (58.8–87.3)	83.9 (74.6–90.2)	34.5 (29.0–40.4)
BT_MAIN_, °C	0.773 (0.697–0.838)	<0.001	≤32.8	72.5 (63.1–80.6)	72.5 (56.1–85.4)	87.8 (81.1–92.3)	49.2 (40.3–58.1)

TTM, targeted temperature management; AUC, area under the curve; CI, confidence interval; PPV, positive predictive value; NPV, negative predictive value; CT_INI_, core temperature at initiation of TTM; BT_INI_, brain temperature at initiation of TTM; CT_MAIN_, core temperature at maintenance phase of TTM; BT_MAIN_, brain temperature at maintenance phase of TTM.

## 4. Discussion

In this study, we examined the relationship between BT and neurological outcomes in OHCA survivors who underwent TTM. We found that both BT_INI_ and BT_MAIN_ were significantly lower in patients with poor outcomes. Multivariable logistic regression analysis demonstrated that both BT_INI_ and BT_MAIN_ were independently associated with poor outcomes, whereas CT_INI_ and CT_MAIN_ were not. The prognostic performance of BT_INI_ and BT_MAIN_ was considered fair.

There have been several attempts to identify the association between body temperature and outcomes in cardiac arrest patients [6, 7, 8, 9, 20]. den Hartog *et al*. [20] reported that spontaneous hypothermia (<35 °C) on intensive care unit admission independently predicts poor neurological outcomes after cardiac arrest, suggesting it may indicate impaired thermoregulation and severe hypoxic-brain injury. Similarly, Benz-Woerner *et al*. [6] found that lower spontaneous body temperature on admission is related with increased in-hospital mortality in comatose cardiac arrest survivors who received TTM. Additionally, prolonged passive rewarming correlated with poor outcomes, possibly indicating impaired thermoregulation [6]. Palka *et al*. [7] demonstrated that spontaneous hypothermia (≤34 °C) on admission is independently associated with early diffuse anoxic brain injury on initial computed tomography scans in post-cardiac arrest patients, suggesting its potential utility as a clinical marker of severe hypoxic brain injury.

Previous studies have demonstrated associations between low CT after ROSC and poor neurological outcomes [6, 7, 20]. However, CT may be influenced more by systemic physiological responses rather than directly reflecting hypoxic-ischemic brain injury. Coppler *et al*. [21] reported that in comatose cardiac arrest survivors, BT is on average 0.34 °C higher than CT and exceeds it by ≥1 °C in 7% of observations, with changes in BT also lagging behind CT by approximately 27 min. Therefore, BT is generally higher than CT, which seems to reflect active brain metabolism. Interestingly, in contrast to previous studies focusing primarily on CT, our findings highlight that a lower BT relative to CT is independently associated with poor outcomes. This relatively low BT may therefore reflect severe metabolic suppression and impaired cerebral perfusion after cardiac arrest.

BT is primarily regulated by the balance between metabolic heat production and heat removal via cerebral blood flow [22]. Cerebral blood flow not only delivers oxygen and nutrients but also dissipates heat generated by neuronal activity, thereby maintaining thermal homeostasis [11]. During cardiac arrest, cerebral perfusion is abruptly interrupted, resulting in a rapid decline in brain metabolic activity [23]. Because the brain has intrinsically high metabolic demands, it depends heavily on a continuous oxygen supply to sustain adenosine triphosphate (ATP) production through oxidative phosphorylation [12, 24]. When oxygen delivery ceases, ATP synthesis is severely impaired. Since cerebral heat production largely depends on oxygen-driven ATP synthesis, decreased metabolic activity after cardiac arrest results in a lower BT [12]. Severe brain injury can disrupt cerebral blood flow, which in turn affects BT regulation. Reduced perfusion may metabolically limit heat production, potentially leading to decreased BT. Zhu *et al*. [25] demonstrated that reduced cerebral blood flow results in lower BT relative to CT, highlighting the dominant role of perfusion in cerebral thermal regulation. In their rat model, different anesthetics were used to modulate cerebral perfusion, and BT was consistently lower than CT when blood flow was reduced [25]. The largest brain–core temperature gradient has been reported under α-chloralose anesthesia, which produces the most profound cerebral hypoperfusion [25]. When hypercapnia was induced to enhance blood flow, BT increased in all groups, and the rate of temperature rise correlated with baseline perfusion levels [25]. These findings suggest that cerebral blood flow stabilizes BT by facilitating heat exchange with the systemic circulation [25].

We acknowledge that the use of a zero-heat-flux sensor (3M™ Bair Hugger™) on the forehead, though clinically practical and noninvasive, presents inherent limitations when compared with invasive intracranial temperature monitoring methods. Prior validation studies comparing invasive intracranial and noninvasive forehead temperature sensors reported generally good agreement under stable physiological conditions, with a mean difference of approximately 0.4 °C [15]. However, during periods of rapid temperature changes, such as induction (–1.1 °C difference) and rewarming (0.7 °C difference), discrepancies were observed to be more pronounced due to thermal inertia and delayed heat conduction through the skin and skull [15]. Therefore, clinicians should interpret readings from a zero-heat-flux sensor with caution, especially during unstable physiological states.

Several limitations should be acknowledged. First, it was a single-center observational study with a relatively small sample size (n = 149), which may limit the generalizability of the findings due to potential institution-specific practices in TTM, patient selection criteria, or regional demographic factors. Second, BT was measured noninvasively using a zero-heat-flux thermometer placed on the forehead, which might not precisely reflect deep intracranial temperatures. Third, although we proposed plausible physiological mechanisms linking lower BT to impaired cerebral metabolism and perfusion, our study did not include comparisons with established prognostic tools, such as neuroimaging, neurophysiological assessments, and serum biomarker measurements, which could have provided more comprehensive insights. Despite the fair prognostic performance of BT (AUC, 0.728–0.773), direct comparisons with these prognostic tools would better clarify its role within a multimodal prognostic framework for cardiac arrest survivors. Fourth, our analysis was limited to patients who received TTM with a target temperature of 33 °C. As a result, we could not determine whether BT serves as a reliable predictor of neurological outcomes across various target temperatures. This limitation is particularly relevant given recent guideline recommendations favoring more individualized TTM strategies (e.g., 33–36 °C). Although a subset of patients (n = 25) in our cohort were treated at temperatures other than 33 °C, the sample size was insufficient for analysis. Future studies involving larger and more heterogeneous patient populations treated with various TTM strategies are warranted to enhance the external validity of our findings. Fifth, we examined neurological outcomes at 6 months after ROSC via structured phone interviews using the CPC scale. Although this method is practical for long-term follow-up, it may be less precise than in-person assessments and susceptible to observer bias, particularly when differentiating between adjacent categories such as CPC 3 (severe disability) and CPC 4 (vegetative state). Finally, this study focused on representative BT values at the initiation of TTM and during the maintenance phase. However, we did not examine temporal trends or fluctuations in BT throughout the entire cooling period. Future studies incorporating continuous BT monitoring may provide additional prognostic insights beyond those offered by static temperature measurements.

## 5. Conclusions

In this study, comatose OHCA patients who received TTM at 33 °C had a lower BT measured at both the initiation and maintenance phases of TTM. These BTs were independently associated with poor neurological outcomes at 6 months after ROSC. BT measurements demonstrated better prognostic value than CT. Our findings suggest that BT could serve as a more precise marker of hypoxic brain injury following cardiac arrest. Further larger-scale, multicenter studies would help confirm these findings and determine the clinical implications of direct BT monitoring during TTM.

## Availability of Data and Materials

All data generated or analyzed during this study are included in this article and its supplementary material files. Further enquiries can be directed to the corresponding author.

## Supplementary Material

Supplementary material associated with this article can be found, in the online version, at https://doi.org/10.31083/RCM43855.
