# Supplementary material for: The Association Between Brain Temperature and Neurological Outcome in Out-of-Hospital Cardiac Arrest Patients Who Received Targeted Temperature Management at 33 °C
Source: Rev Cardiovasc Med. 2025 Nov 18;26(11):43855. doi: 10.31083/RCM43855 (PMC12680982; doi:10.31083/RCM43855)
Supplement: Supplementary file 1 [file 2153-8174-26-11-43855-s1.docx]

Supplementary Fig. 1. Interview algorithm used in our hospital to determine neurological prognosis after return of spontaneous circulation


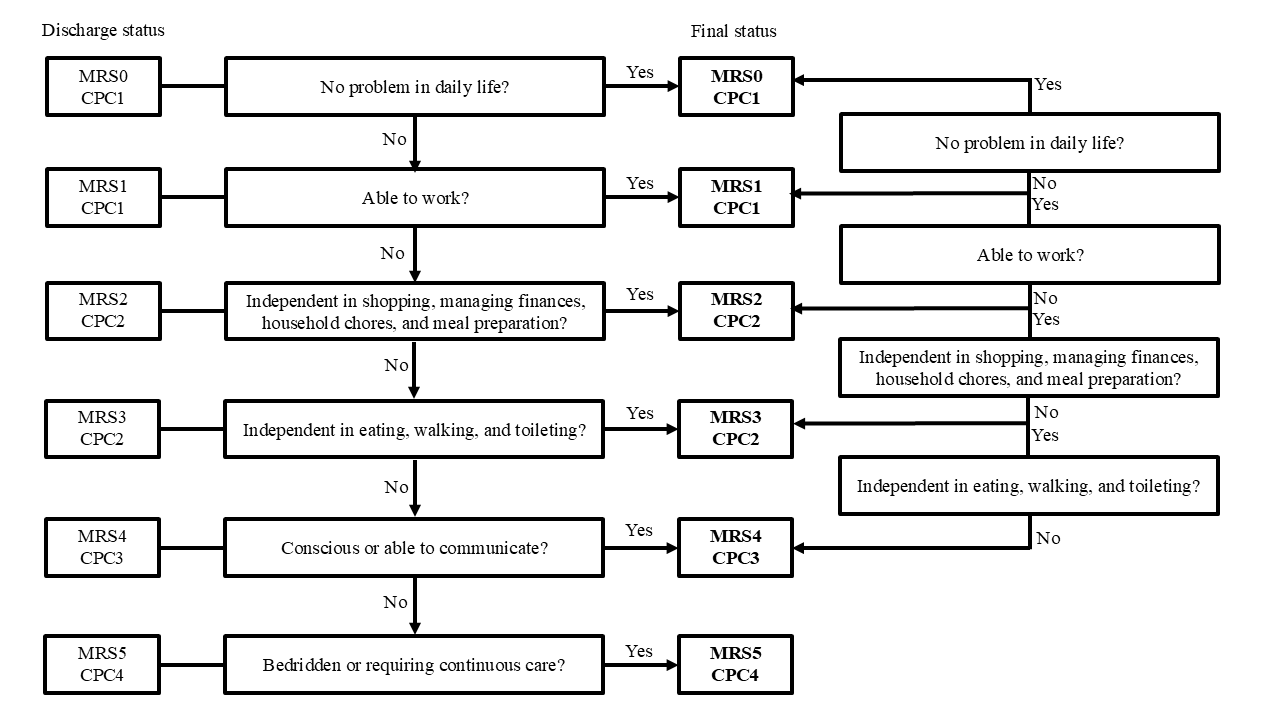


Supplementary Table 1. Multivariable analysis of variables associated with poor neurological outcomes at 6 months.

|  | Adjusted OR (95% CI) | p |  | Adjusted OR (95% CI) | p |
| --- | --- | --- | --- | --- | --- |
| A. Step 1 |  |  | B. Step 2 |  |  |
| Age, years | 1.089 (1.027–1.155) | <0.004 | Age, years | 1.089 (1.028–1.155) | 0.004 |
| Diabetes | 1.549 (0.291–8.244) | 0.608 | Diabetes | 1.534 (0.293–8.040) | 0.613 |
| Renal impairment | 0.797 (0.056–11.236) | 0.866 | Renal impairment | 0.804 (0.057–11.385) | 0.872 |
| Witnessed collapse | 0.601 (0.115–3.498) | 0.601 | Witnessed collapse | 0.658 (0.146–2.958) | 0.585 |
| Bystander CPR | 1.077 (0.206–5.637) | 0.930 | Shockable rhythm | 0.020 (0.001–0.289) | 0.004 |
| Shockable rhythm | 0.020 (0.001–0.288) | 0.004 | Cardiac etiology | 1.375 (0.083–22.911) | 0.824 |
| Cardiac etiology | 1.390 (0.083–23.218) | 0.819 | Time to ROSC, min | 1.077 (1.024–1.133) | 0.004 |
| Time to ROSC, min | 1.077 (1.024–1.133) | 0.004 | Lactate, mmol/L | 1.170 (0.986–1.388) | 0.073 |
| Lactate, mmol/L | 1.172 (0.984–1.395) | 0.076 | Glucose, mg/dL | 0.996 (0.991–1.000) | 0.054 |
| Glucose, mg/dL | 0.996 (0.991–1.000) | 0.055 | PaO_2_, mmHg | 1.001 (0.996–1.007) | 0.662 |
| PaO_2_, mmHg | 1.001 (0.996–1.007) | 0.659 | PaCO_2_, mmHg | 1.022 (0.971–1.075) | 0.411 |
| PaCO_2_, mmHg | 1.022 (0.971–1.075) | 0.411 | CT_INI_, ℃ | 3.308 (0.857–12.775) | 0.083 |
| CT_INI_, ℃ | 3.304 (0.851–12.825) | 0.084 | BT_INI_, ℃ | 0.212 (0.050–0.897) | 0.035 |
| BT_INI_, ℃ | 0.212 (0.050–0.901) | 0.036 | CT_MAIN_, ℃ | 0.310 (0.000–279.502) | 0.736 |
| CT_MAIN_, ℃ | 0.301 (0.000–329.593) | 0.737 | BT_MAIN_, ℃ | 0.103 (0.015–0.695) | 0.020 |
| BT_MAIN_, ℃ | 0.102 (0.015–0.698) | 0.020 |  |  |  |
|  |  |  |  |  |  |
| C. Step 3 |  |  | D. Step 4 |  |  |
| Age, years | 1.089 (1.028–1.153) | 0.004 | Age, years | 1.090 (1.030–1.154) | 0.003 |
| Diabetes | 1.481 (0.301–7.276) | 0.629 | Diabetes | 1.506 (0.307–7.377) | 0.614 |
| Witnessed collapse | 0.669 (0.151–2.959) | 0.596 | Witnessed collapse | 0.693 (0.161–2.984) | 0.622 |
| Shockable rhythm | 0.020 (0.001–0.289) | 0.004 | Shockable rhythm | 0.025 (0.004–0.159) | <0.001 |
| Cardiac etiology | 1.397 (0.084–23.241) | 0.816 | Time to ROSC, min | 1.077 (1.024–1.133) | 0.004 |
| Time to ROSC, min | 1.077 (1.024–1.133) | 0.004 | Lactate, mmol/L | 1.171 (0.987–1.389) | 0.069 |
| Lactate, mmol/L | 1.171 (0.988–1.388) | 0.068 | Glucose, mg/dL | 0.996 (0.991–1.000) | 0.057 |
| Glucose, mg/dL | 0.996 (0.991–1.000) | 0.055 | PaO_2_, mmHg | 1.001 (0.995–1.007) | 0.691 |
| PaO_2_, mmHg | 1.001 (0.996–1.007) | 0.672 | PaCO_2_, mmHg | 1.020 (0.971–1.072) | 0.431 |
| PaCO_2_, mmHg | 1.021 (0.971–1.074) | 0.419 | CT_INI_, ℃ | 3.401 (0.898–12.885) | 0.072 |
| CT_INI_, ℃ | 3.372 (0.894–12.723) | 0.073 | BT_INI_, ℃ | 0.208 (0.049–0.879) | 0.033 |
| BT_INI_, ℃ | 0.209 (0.050–0.877) | 0.032 | CT_MAIN_, ℃ | 0.325 (0.001–142.846) | 0.717 |
| CT_MAIN_, ℃ | 0.340 (0.001–168.258) | 0.733 | BT_MAIN_, ℃ | 0.104 (0.016–0.675) | 0.018 |
| BT_MAIN_, ℃ | 0.101 (0.015–0.669) | 0.017 |  |  |  |
|  |  |  |  |  |  |
| E. Step 5 |  |  | F. Step 6 |  |  |
| Age, years | 1.088 (1.028–1.151) | 0.004 | Age, years | 1.084 (1.027–1.143) | 0.003 |
| Diabetes | 1.575 (0.325–7.632) | 0.573 | Diabetes | 1.709 (0.351–8.316) | 0.507 |
| Witnessed collapse | 0.672 (0.158–2.859) | 0.591 | Shockable rhythm | 0.024 (0.004–0.150) | <0.001 |
| Shockable rhythm | 0.025 (0.004–0.161) | <0.001 | Time to ROSC, min | 1.077 (1.024–1.133) | 0.004 |
| Time to ROSC, min | 1.077 (1.023–1.133) | 0.004 | Lactate, mmol/L | 1.158 (0.979–1.369) | 0.087 |
| Lactate, mmol/L | 1.168 (0.982–1.388) | 0.079 | Glucose, mg/dL | 0.996 (0.992–1.000) | 0.071 |
| Glucose, mg/dL | 0.996 (0.991–1.000) | 0.055 | PaCO_2_, mmHg | 1.017 (0.969–1.066) | 0.500 |
| PaCO_2_, mmHg | 1.017 (0.970–1.066) | 0.487 | CT_INI_, ℃ | 3.324 (0.895–12.352) | 0.073 |
| CT_INI_, ℃ | 3.551 (0.927–13.609) | 0.065 | BT_INI_, ℃ | 0.215 (0.051–0.901) | 0.036 |
| BT_INI_, ℃ | 0.203 (0.047–0.872) | 0.032 | CT_MAIN_, ℃ | 0.239 (0.000–282.824) | 0.692 |
| CT_MAIN_, ℃ | 0.235 (0.000–374.566) | 0.700 | BT_MAIN_, ℃ | 0.115 (0.018–0.740) | 0.023 |
| BT_MAIN_, ℃ | 0.111 (0.017–0.707) | 0.020 |  |  |  |
|  |  |  |  |  |  |
| G. Step 7 |  |  | H. Step 8 |  |  |
| Age, years | 1.084 (1.029–1.142) | 0.003 | Age, years | 1.086 (1.032–1.144) | 0.002 |
| Diabetes | 1.702 (0.349–8.292) | 0.510 | Shockable rhythm | 0.028 (0.005–0.153) | <0.001 |
| Shockable rhythm | 0.027 (0.005–0.155) | <0.001 | Time to ROSC, min | 1.081 (1.028–1.137) | 0.002 |
| Time to ROSC, min | 1.077 (1.023–1.133) | 0.004 | Lactate, mmol/L | 1.145 (0.987–1.328) | 0.074 |
| Lactate, mmol/L | 1.145 (0.984–1.332) | 0.081 | Glucose, mg/dL | 0.996 (0.992–1.000) | 0.061 |
| Glucose, mg/dL | 0.996 (0.992–1.000) | 0.071 | PaCO_2_, mmHg | 1.019 (0.974–1.067) | 0.415 |
| PaCO_2_, mmHg | 1.020 (0.974–1.069) | 0.401 | CT_INI_, ℃ | 3.043 (0.837–11.068) | 0.091 |
| CT_INI_, ℃ | 3.052 (0.850–10.959) | 0.087 | BT_INI_, ℃ | 0.230 (0.056–0.936) | 0.040 |
| BT_INI_, ℃ | 0.234 (0.058–0.944) | 0.041 | BT_MAIN_, ℃ | 0.083 (0.019–0.360) | <0.001 |
| BT_MAIN_, ℃ | 0.082 (0.019–0.364) | <0.001 |  |  |  |
|  |  |  |  |  |  |
| I. Step 9 |  |  |  |  |  |
| Age, years | 1.088 (1.034–1.145) | <0.001 |  |  |  |
| Shockable rhythm | 0.023 (0.004–0.120) | <0.001 |  |  |  |
| Time to ROSC, min | 1.083 (1.030–1.139) | 0.002 |  |  |  |
| Lactate, mmol/L | 1.150 (0.993–1.332) | 0.062 |  |  |  |
| Glucose, mg/dL | 0.996 (0.992–1.000) | 0.063 |  |  |  |
| CT_INI_, ℃ | 3.193 (0.860–11.857) | 0.083 |  |  |  |
| BT_INI_, ℃ | 0.223 (0.054–0.917) | 0.038 |  |  |  |
| BT_MAIN_, ℃ | 0.078 (0.019–0.322) | <0.001 |  |  |  |

As the backward stepwise selection progressed, the following variables were sequentially removed from the model:

bystander CPR, renal impairment, cardiac etiology, PaO_2_, witness collapse, CT_MAIN_, diabetes, and PaCO_2_

OR, odds ratio; CI, confidence interval; CPR, cardiopulmonary resuscitation; ROSC, return of spontaneous circulation; PaO_2_, partial pressure of oxygen; PaCO_2_, partial pressure of carbon dioxide.
